# Laparoscopic resection of a cecal carcinoma with a Retzius vein shunt: A case report

**DOI:** 10.1097/MD.0000000000042456

**Published:** 2025-05-16

**Authors:** Yusuke Kono, Manabu Yamamoto, Chiharu Yasui, Ryo Ishiguro, Takuki Yagyu, Kyoichi Kihara, Tomoyuki Matsunaga, Shuichi Takano, Naruo Tokuyasu, Teruhisa Sakamoto, Toshimichi Hasegawa, Yoshiyuki Fujiwara

**Affiliations:** a Department of Surgery, Division of Gastrointestinal and Pediatric Surgery, Faculty of Medicine, Tottori University, Yonago, Japan.

**Keywords:** cecal cancer, portosystemic shunt, Retzius shunt

## Abstract

**Rationale:**

A Retzius shunt between the ileocecal vein and inferior vena cava is rare. To avoid major hemorrhage due to shunt injury, it is essential to evaluate vascular abnormalities on preoperative imaging and to confirm anatomic structures in detail during the operation.

**Patient concerns:**

A 71-year-old woman with diabetes and asthma developed anaemia.

**Diagnoses:**

She was diagnosed with cecal cancer by endoscopy and found to have a venous malformation forming a Retzius shunt from the ileocecal vein to the inferior vena cava on computed tomography.

**Intervention:**

Laparoscopic ileocecal resection was performed. Colonic mobilization and resection of the ileocecal vessels were performed while minimizing traction on the shunt. After confirming the anatomy, the Retzius shunt was resected without complications.

**Outcomes:**

The patient experienced an unremarkable postoperative clinical course without complications.

**Lessons:**

This case involved a rare vascular anomaly associated with colorectal cancer, characterized by an abnormal blood vessel connecting the inferior vena cava and mesenteric veins. When abnormal vessels are detected on preoperative abdominal computed tomography, a Retzius shunt should be considered. Detailed review of the imaging, careful surgical manipulation to avoid shunt damage, and thorough anatomical verification, are important to perform a safe operation.

## 1. Introduction

A Retzius shunt is a rare retroperitoneal venous anastomosis between the portal vein and vena cava.^[1,2]^ Because injury of a Retzius shunt during surgery can result in major hemorrhage, it is essential to fully evaluate vascular abnormalities on preoperative imaging and to confirm anatomic structures in detail during the operation. We report a cecal carcinoma in a patient associated with an ileocecal vein–inferior vena cava shunt via a gonadal vein which was safely resected using laparoscopic technique.

## 2. Case report

**Figure 1.**
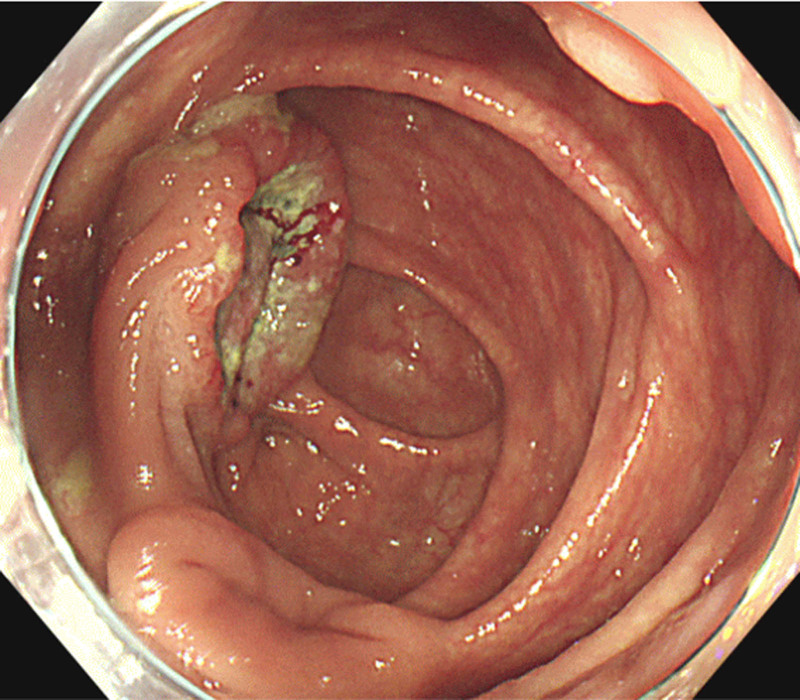
Colonoscopy revealed a type 2 tumor localized to the cecum.

**Figure 2.**
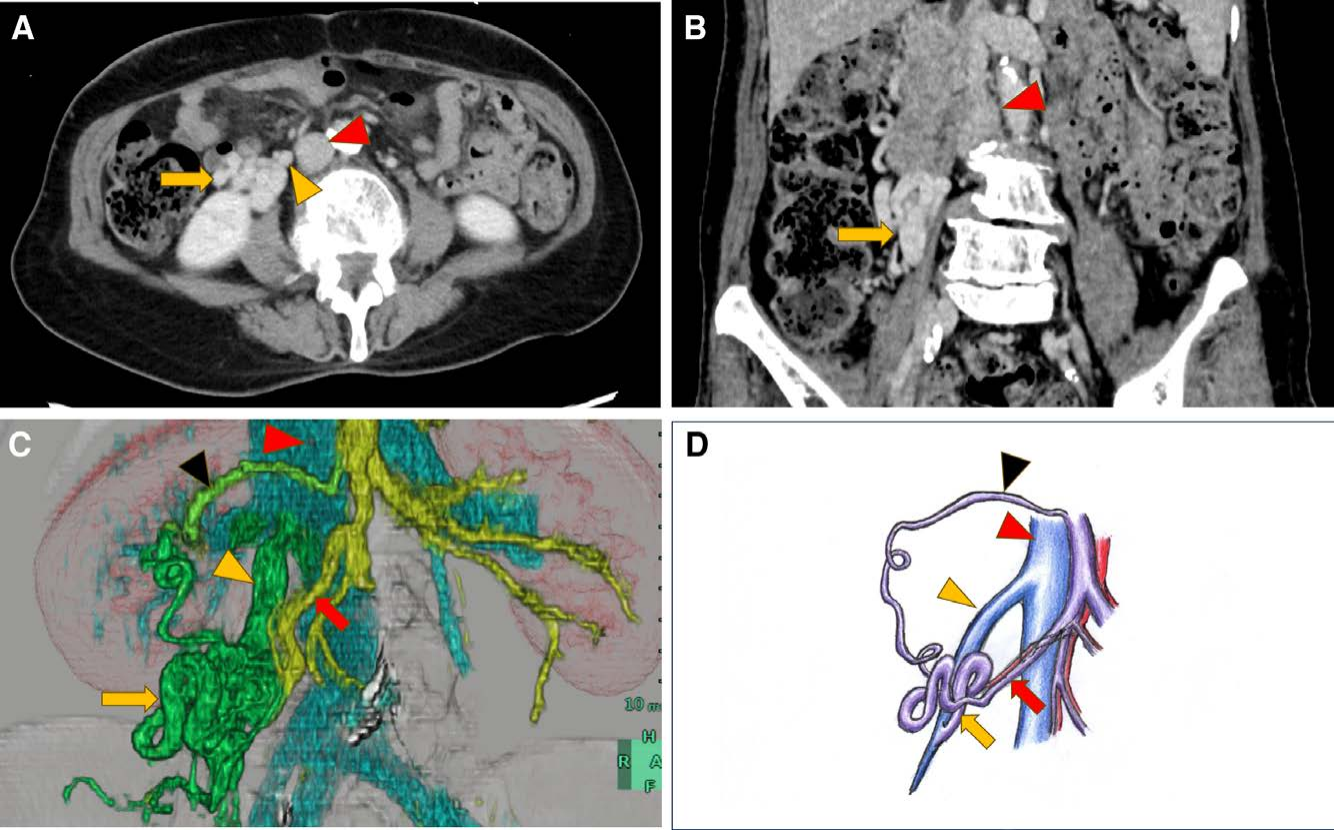
Axial (A), coronal (B), and 3-dimensional reconstruction (C) views of contrast-enhanced computed tomography. A schematic diagram of the vascular structure (D). They showed a Retzius shunt (yellow arrow) connecting to the inferior vena cava (red arrowhead) via a dilated gonadal vein (yellow arrowhead) in the ileocecal area. Ileocecal vein (red arrow) communicates with a cluster of dilated vessels. The dilated vein in the right colic region, continuous with the dilated vessels (black arrowhead), communicates with the superior mesenteric vein. CT = computed tomography.

A 71-year-old woman with a history of diabetes and asthma was diagnosed with cecal cancer after an anemia workup. Her surgical history was notable for a total abdominal hysterectomy and bilateral salpingo-oophorectomy performed over 20 years previously. Blood testing showed no specific abnormal findings. Colonoscopy showed a type 2 tumor at the cecum (biopsy histology was moderately differentiated adenocarcinoma); no varicose veins were found on the mucosal surface (Fig. 1). Contrast-enhanced computed tomography (CT) showed multiple dilated vessels in the ileocecal area. These vessels were continuous with the inferior vena cava via a dilated gonadal vein which had been resected and was blind ended (Fig. 2A–D). The ileocecal vein communicates with a cluster of dilated vessels, which are continuous with the dilated veins in the right colic region and communicate with the superior mesenteric vein (Fig. 2C, D). Laparoscopic ileocecal resection of the cecal adenocarcinoma was performed.

**Figure 3.**
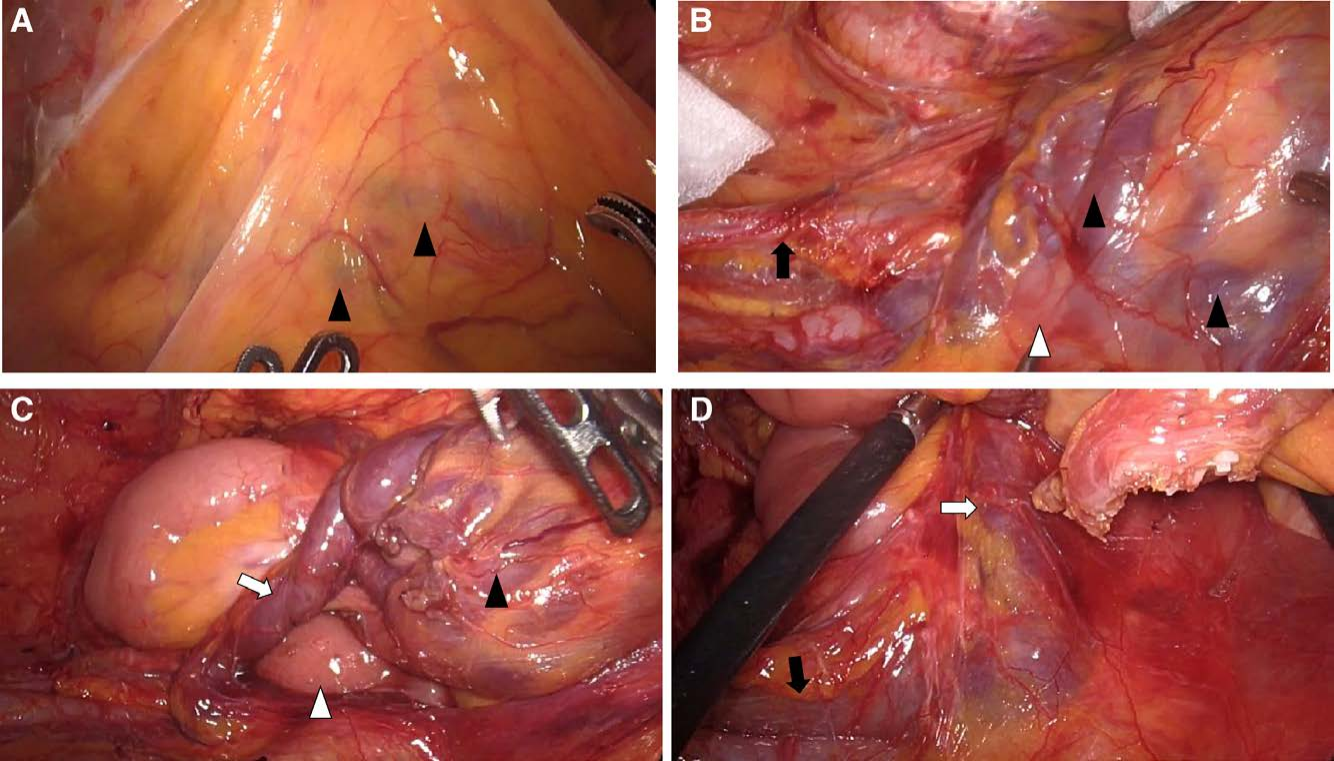
Intraoperative findings. (A, B) Dilated vessels (black arrowhead) were seen within the ileal mesentery (gonadal vein, black arrow; duodenum, white arrow). (C) A Retzius vein (white arrow) communicated from the mesentery to the retroperitoneum; dilated vessels were also seen within the mesentery (duodenum, white arrowhead). (D) The Retzius vein (white arrow) communicated with a gonadal vein (black arrow).

During the operation, the liver appeared normal with a smooth surface. A dilated abnormal vessel was observed within the ileocecal mesentery. After the retroperitoneum was dissected (Fig. 3A, B), colonic mobilization and resection of the ileocecal vessels were performed to confirm the anatomy while minimizing traction on the abnormal vessels. Once we ensured the shunt was a single vessel communicating with the ovarian vein, it was safely resected (Fig. 3C, D). Then, the intestinal tract was resected and anastomosed extraperitoneally. The operation time was 194 minutes. Blood loss volume was 4 mL. The patient’s postoperative course was unremarkable and she was discharged on postoperative day 7. The tumor was staged as T3N0M0 (stage IIA). There were no obvious blood test abnormalities or complications at the 1- and 3-month follow-ups.

## 3. Discussion

Several theories regarding the cause of portosystemic shunts have been proposed. The congenital origin theory postulates that connection between the caval and portal systems persists throughout embryologic development. In contrast, the acquired theory points to portal hypertension associated with cirrhosis and/or intra-abdominal adhesions resulting from trauma or surgery.^[2–5]^ In this case, the absence of a history of liver cirrhosis and findings of portal hypertension makes the involvement of liver cirrhosis unlikely. Ibukuro et al reviewed 130 patients who underwent CT during arterial portography and found a Retzius shunt of the ileocecal vein in half of them.^[6]^ However, they included microvessels, which are not problematic at the time of surgery and are probably treated with ultrasonically activated devices or electrocautery without problems. In addition, the Retzius shunt prevalence did not differ between patients with and without cirrhosis. This fact and the absence of patients with cirrhosis in other previous case reports suggest that cirrhosis may not be involved in Retzius shunt formation.^[7–9]^ Even if a congenital shunt is present, it is considered that an increase in venous pressure is necessary for the shunt to become problematic during surgery. Furukawa et al reported a pathologically proven Retzius shunt associated with an arteriovenous malformation (AVM).^[9]^ In our patient, the traffic between the ileocecal artery and vein was not evident on imaging and a pathological evaluation of the vessels was not conducted; however, similar to the findings of Furukawa et al, the presence of clusters of dilated and tortuous abnormal vessels suggested the possibility of shunt formation due to localized portal hypertension associated with AVM. Arteriovenous shunts in the portal circulation system are most commonly caused by traumatic or iatrogenic factors, accounting for approximately 60% of cases,^[10]^ with the mechanism of occurrence often attributed to mesenteric arteriovenous injury during abdominal surgery.^[11,12]^ This case had remotely undergone a total abdominal hysterectomy and bilateral salpingo-oophorectomy. Unfortunately, we were unable to obtain any information regarding the details or surgical findings of that operation. However, it is possible that the oophorectomy may have contributed to AVM formation resulting in dilated Retzius shunt formation. There are 3 possible mechanisms for the formation of the abnormal vessels in this case. First, the congenital Retzius shunt involving the colonic and ovarian veins may have been dilated due to increased venous pressure caused by the formation of an AVM as a result of vascular injury during previous surgical procedures. Second, both the AVM and Retzius shunt were formed due to vascular injury during previous surgical manipulations. Third, an AVM was formed due to vascular injury during surgical manipulation, and a Retzius shunt with the nearby ovarian vein was gradually formed due to increased venous pressure. Based on previous reports,^[6,9,10]^ the possibility of the first mechanism is considered to be the most likely.

In cases of colorectal cancer with Retzius shunt formation in the dominant vessel, hematogenous metastasis to the lungs via the systemic circulation should be readily expected. Although our patient had no evidence of pulmonary nor hepatic metastases, careful surveillance for lung metastases is warranted. Hara et al reported a patient with rectal cancer and a Retzius shunt as well as isolated lung metastasis; pulmonary resection was performed after resection of the rectal tumor.^[7]^

When a Retizus shunt is to be encountered during surgery, it is extremely important to assess the condition of the vessels before and during the operation because of the possibility of major hemorrhage. In previous reports, vessels have been safely resected by examining the vessels in detail using preoperative 3-dimensional CT and taking precautions during surgery.^[7–9]^ We also performed CT with 3-dimensional reconstructions to examine the shunt and associated vessels in detail. During surgery, the tumor and shunt were safely resected after confirming the anatomy and paying careful attention to avoid traction and other maneuvers which could have resulted in hemorrhage. When abnormal vessels are found on preoperative imaging, it is important to evaluate them in detail, keeping in mind the possibility of a Retzius shunt.

## 4. Conclusion

To our knowledge, this is the 1st report of resection of a right-sided colon cancer associated with a Retzius shunt. Careful preoperative and intraoperative evaluation of the abnormal vessels and their association with adjacent structures is important for safe resection of the tumor and shunt.

## Acknowledgments

We thank Edanz (https://jp.edanz.com/ac) for editing a draft of this manuscript.

## Author contributions

**Writing – original draft:** Yusuke Kono.

**Writing – review & editing:** Manabu Yamamoto, Chiharu Yasui, Ryo Ishiguro, Takuki Yagyu, Kyoichi Kihara, Tomoyuki Matsunaga, Shuichi Takano, Naruo Tokuyasu, Teruhisa Sakamoto, Toshimichi Hasegawa, Yoshiyuki Fujiwara.
